# A Cloud-Based Virtual Outpatient Clinic for Patient-Centered Care: Proof-of-Concept Study

**DOI:** 10.2196/10135

**Published:** 2018-09-24

**Authors:** Jelske Marije de Jong, Paula AM Ogink, Carin GM van Bunningen, Rieke JB Driessen, Lucien JLPG Engelen, Barend Heeren, Sebastian JH Bredie, Tom H van de Belt

**Affiliations:** 1 Radboud REshape Innovation Center Radboud University Medical Center Nijmegen Netherlands; 2 Department of Internal Medicine Radboud University Medical Center Nijmegen Netherlands; 3 Department of Dermatology Radboud University Medical Center Nijmegen Netherlands

**Keywords:** cloud service, digital health, eHealth, mHealth, mobile phone, outpatient clinic, patient-centeredness

## Abstract

**Background:**

Most electronic health (eHealth) interventions offered to patients serve a single purpose and lack integration with other tools or systems. This is problematic because the majority of patients experience comorbidity and chronic disease, see multiple specialists, and therefore have different needs regarding access to patient data, communication with peers or providers, and self-monitoring of vital signs. A multicomponent digital health cloud service that integrates data sharing, collection, and communication could facilitate patient-centered care in combination with a hospital patient portal and care professionals.

**Objective:**

This study aimed to assess the feasibility and functionality of a new cloud-based and multicomponent outpatient clinic, the “Virtual Outpatient Clinic” (VOC).

**Methods:**

The VOC consists of 6 digital tools that facilitate self-monitoring (blood pressure, weight, and pain) and communication with peers and providers (chat and videoconferencing) connected to a cloud-based platform and the hospital patient portal to facilitate access to (self-collected) medical data. In this proof-of-concept study, 10 patients from both Departments of Internal Medicine and Dermatology (N=20) used all options of the VOC for 6 weeks. An eNurse offered support to participants during the study. We assessed the feasibility, usage statistics, content, adherence, and identified technical issues. Moreover, we conducted qualitative interviews with all participants by following a standard interview guide to identify user experiences, including barriers, facilitators, and potential effects.

**Results:**

Most participants successfully used all options of the VOC and were positive about different tools and apps and the integral availability of their information. The adherence was 37% (7/19) for weight scale, 58% (11/19) for blood pressure monitor, and 70% (14/20) and 85% (17/20) for pain score and daily questions, respectively. The adherence for personal health record was 65% (13/20) and 60% (12/20) for the patient portal system. Qualitative data showed that performance and effort expectancy scored high among participants, indicating that using the VOC is convenient, easy, and time-saving.

**Conclusions:**

The VOC is a promising integrated Web-based technology that combines self-management, data sharing, and communication between patients and professionals. The system can be personalized by connecting various numbers of components, which could make it a relevant tool for other patient groups. Before a system, such as the VOC, can be implemented in daily practice, prospective studies focused on evaluating outcomes, costs, and patient-centeredness are needed.

## Introduction

Digital technology is transforming health care. Electronic health (eHealth) and mobile health (mHealth) technologies (Textbox 1) facilitate self-management (eg, self-monitoring of weight [1], increase medication adherence [2,3], and promote diabetes self-management [4]), (tele-) communication (eg, in self-management of hypertension [5], ambulatory care for chronic diseases [6], and patient-doctor communication [7]), efficient data sharing (eg, using a personal health record (PHR) [8]), and remote monitoring (eg, remote pain assessment [9,10]). Although evidence on their efficacy remains limited in some sectors [11,12], more robust evidence is already available in others [13,14].

Most eHealth and mHealth solutions offered to patients serve a single purpose or have been developed for use within one medical specialty, for example, teledermatology to reduce face-to-face consultations, or self-management of hypertension by self-titration of medication [15,16]. Since many patients are comorbid and therefore visit multiple medical specialists [17,18], multipurpose and integrated systems could be an efficient way to further improve the quality of care. Particularly, considering a variety of single-purpose apps may increase the nonadherence or attrition rates because of the time invested in using multiple apps outweighs the benefits [19]. An example of such a multipurpose system has been described by Alnosayan et al, which was provided to patients with heart failure for support after discharge [20]. Both patients and nurses regularly used the tools, and patients showed above average satisfaction with the system.

The majority of hospitals and individual health care providers use electronic medical records (EMRs) to store patients’ medical data. In some cases, patients have online access to their diagnoses, medication, or lab results. However, patient access is often not possible because of formal obstructions or technical barriers, or because patients are simply unaware of the possibility [25]. Moreover, some health care providers refuse to give patients access to the EMR because it contains clinical notes [26,27]. The lack of patient access seems to be a missed opportunity, as the benefits of patient access have been well described [28]. A proposed solution is a hospital’s patient portal system (PPS) that is part of the EMR and presents a selection of information such as lab results, appointments, and medication. Most health care providers share a positive attitude toward a PPS [26,27]. The same goes for adult and pediatric patients, who are primarily positive about such a system [29,30]. Another way of providing patients with their data is the use of PHRs; these are comparable to a PPS but exist separately from the EMR and are owned and fully controlled by patients. Patients already use PHRs because it is easy to access their health data, self-manage disease, and have a more productive communication with their health care provider [31-34]. Physicians who already use electronic communication perceive it as convenient, time-saving, efficient, and safe [35]. The use of a secure electronic messaging system showed a reduction in the number of office visits [36]. Overall, both PPS and PHR could be efficient ways for patients to collect, present, and share health data.

Regarding the need for integrated self-monitoring and self-managing systems, we designed a state-of-the-art integrated multicomponent digital health cloud service, the “Virtual Outpatient Clinic” (VOC). The VOC is a combination of PPS and PHR and consists of multiple health-monitoring tools, in which data storage and presentation are integrated and can be accessed by both patients and health care professionals. This study aims to assess the feasibility and functionality of the VOC.

**Definition of eHealth and mHealth.**

**eHealth and mHealth**

The term eHealth, together with related terms like mHealth, Health 2.0, telecare, and telemedicine, has gained popularity over the last 20 years [21]. However, different definitions exist [22]. The most commonly used definition is Eysenbach’s definition: “e-health is an emerging field in the intersection of medical informatics, public health and business, referring to health services and information delivered or enhanced through the Internet and related technologies. In a broader sense, the term characterizes not only a technical development, but also a state-of-mind, a way of thinking, an attitude, and a commitment for networked, global thinking, to improve health care locally, regionally, and worldwide by using information and communication technology.” [23]. For mHealth, the World Health Organization stated that there is no common definition, but that it can be considered a part of eHealth. The World Health Organization defined mHealth as “medical and public health practice supported by mobile devices, such as smartphones, patient monitoring devices, personal digital assistants, and other wireless devices” [24].

## Methods

### Eligibility

In this proof-of-concept study, patients treated at the Departments of Internal Medicine and Dermatology of a university medical center in the Netherlands were invited to use and evaluate the VOC. These patients were selected because their disease spectrum was broad with various comorbidities. There were no limitations regarding (co)morbidity. Adult participants were approached between December 2016 and February 2017 and were found eligible if they owned a smartphone with mobile internet access. The inclusion was based on the “first come, first serve” principle; nurses invited patients until 10 patients of both departments participated in the study, which lasted 6 weeks. An eNurse supported participants with the initial set-up and during the study. The study protocol was reviewed and approved to proceed by the local Medical Ethical Committee (ID: 2016-2990). All participants provided signed informed consent.

### Virtual Outpatient Clinic

**Figure 1:**
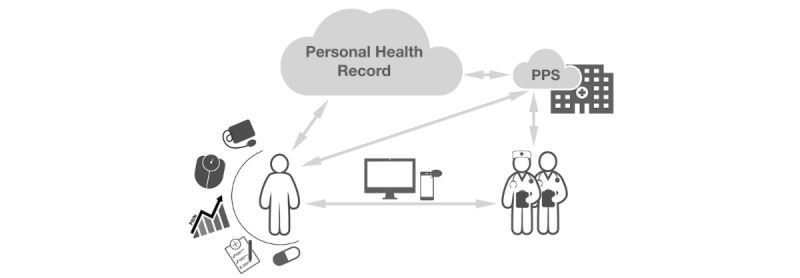
Overview of the Virtual Outpatient Clinic design, with its 3 main components. PPS: patient portal system.

The VOC (Figure 1) consists of 2 measuring devices and 4 smartphone apps, of which 2 collected health data and 2 facilitated communication. To centralize the data collection and facilitate patient access, a PHR and a PPS were used. The Patients Know Best (PKB) platform was selected as the PHR to present health-monitoring data collected during the study period [37]. Figure 1 shows that the patient uses eHealth and mHealth tools to monitor the health status. The patient can communicate with the eNurse using a secure messenger app and videoconferencing tool. Measurements performed by the patient are saved in a PHR and a PPS of the EMR, which the patient can visit at any time. In addition, the team of health care professionals has access to the PPS and PHR. Data can be exchanged between the PHR and the PPS. Multimedia Appendices 1 and 2 present the screenshots of PKB. It has been designed to allow patients to manage their health data and share medical data with their physicians, link multiple health-related apps and devices to PKB to synchronize data, and communicate with health care providers. Data from 4 eHealth tools were automatically stored (real-time) in PKB (Table 1). The PPS used was a modified variant of Epic’s MyChart (Dutch language and layout in the hospital’s style) to facilitate communication between patients and physicians and to present medical test results and other medical information. Measurement results of the weighing scale and blood pressure device were collected in both PPS and PHR (Table 1).

In total, 6 eHealth tools were used. In addition, 2 measuring devices including a blood pressure monitor (BPM; Withings BPM; Nokia Health, Espoo, Finland) and a weighing scale (Withings Body+; Nokia Health) to monitor blood pressure and heart rate and weight, body fat, and water percentage, respectively. Withings devices were connected with a smartphone app to store measurements in addition to the platforms. The remaining 4 tools were all smartphone apps. To facilitate videoconferences with the eNurse, the tool “FaceTalk” (QConferencing, Amsterdam, the Netherlands) was used, which could be used on any device or computer. The second communication tool was “Kanta” (Topicus zorg, Deventer, the Netherlands), a secured messenger app. The questionnaire tool “Q1.6” (Questions.ai, Antwerp, Belgium) was used to monitor patients’ actual status by the Dermatology Life Quality Index (DLQI) and the level of pain with a visual analog scale (VAS) score. The questionnaires were selected because they are commonly used in these departments. The DLQI was most applicable to patients of the Department of Dermatology and focused on the physical symptoms of the skin and how the skin problems affected their daily life.

**Table 1:** Protocol for participants on using different tools and platforms during the study period (6 weeks).

Tool or platform	Instruction	Expected user statistics per participant
Withings blood pressure monitor^a,b^	Measure blood pressure and heart rate (2 periods of 5 consecutive days)	10 measurements
Withings body+^a,b^	Measure weight (2 times a week)	12 measurements
Q1.6^a^	Fill out pain VAS^c^ score (daily); fill out DLQI^d^ (every 2 weeks)	42 pain VAS scores and 3 completed DLQIs
MedApp^a^	Enter current medication use (if applicable)	Medication list
Kanta	Contact eNurse for technical or logistic support	Total number of conversations and overview of topics
FaceTalk	Have one digital meeting with eNurse	Number of successful digital meetings
Personal health record	Log in once weekly to review data	6 log-ins
Patient portal system	Log in once weekly to review data	6 log-ins

^a^Real-time data presented in personal health record.

^b^Real-time data presented in the patient portal system.

^c^VAS: visual analog scale.

^d^DLQI: Dermatology Life Quality Index.

Conversely, the VAS score for pain was applicable to patients from both departments. Pain scores were used to assess patients’ situation, for example, during recovery at home. Notifications on medication intake were sent by “MedApp” (PharmIT, Eindhoven, the Netherlands) to keep track of medication compliance and inventory; medication could be entered by scanning the barcode on the package.

### Study Procedures

The 2 eNurses (one in each department), with at least 5 years of experience as a nurse in the specific department’s outpatient clinic, supported participants during the study. They were familiar with the tools and platforms, answered questions from participants, and were allowed to contact individual participants through the communication tools and offer support. After being informed about the study and signing informed consent, all participants were informed about the tools by the eNurse. In addition, the eNurse assisted in downloading the apps on their personal smartphone, installing the devices, and linking tools to platforms if necessary. Participants were asked to use the tools and perform several measurements for 6 weeks according to the provided protocol. Table 1 summarizes the protocol with the expected user numbers per participant at the end of the study period when a participant adhered completely to the protocol. This schedule was selected on the basis of recommendations of a medical specialist and represented the real-life situation. Both participants and eNurses kept predesigned logbooks, with detailed instructions for the use of the various tools and platform during the study period. As a compensation for their participation, participants could keep the Withings tools after the study.

### Evaluation

We assessed the log-in data, content, and users’ experiences to determine the VOC feasibility. User statistics of tools and platforms were collected by 2 researchers (JMJ and PAMO), using the PHR platform, PPS platform, Q1.6 dashboard, eNurse logbooks, and participant logbooks. Starting dates were extracted from the logbooks participants handed in; if a logbook was not present or no dates were indicated, the starting date was established as the date the first use of an app or device was registered. In addition, users’ experiences were assessed by semistructured interviews with individual participants by 2 researchers (THB and T Chau). Interviews took place at the hospital and were scheduled on the last day of the 6-week study or shortly after to ensure that all patients were able to thoroughly test all features. To fully capture the main elements related to acceptance of technology, the interview guide was designed according to the Unified Theory of Acceptance and Use of Technology 2 (UTAUT2) interview framework [38]. This framework consists of the themes performance expectancy, effort expectancy, social influence, and facilitating conditions. In addition to these themes, support, safety and privacy, technology, and routine regarding the VOC were discussed during the interview. The interview guide is available on request.

### Analysis

#### Logging Data and Content

We used IBM SPSS version 22 (IBM Analytics, New York) to analyze quantitative data. The normality of the data distribution was assessed using the Shapiro-Wilk normality test. Normally distributed data are presented as mean (SD), and nonnormally distributed data are presented as median and interquartile range (IQR: 25-75). In addition, user statistics of tools and platforms were determined and compared with the expected user statistics. We assessed the technical feasibility of the VOC by assessing its use and by experiences, including barriers and facilitators. As there are no official criteria for the feasibility [39], we reasoned that 100% adherence would be impossible as technical problems often occur with new digital platforms. Moreover, we expected that the platform would not work for everyone, as personal preferences are unique. Therefore, we set the criterion for the feasibility to 80% adherence, meaning that 80% of all measurements, such as blood pressure measurements, were successfully performed and are available in the Web-based system. Moreover, the technical feasibility was assessed for the Withings Body+ weight scale, Withings BPM, Q1.6, PHR, and PPS. For Kanta, the number and content of messages sent were determined. Medication was entered in MedApp, and actual prescriptions were compared. The number of FaceTalk appointments was assessed, and failures were reported. The number of successful links of tools to a platform was determined, and causes for unsuccessful links were reported. Problems with tools were reported.

#### Experiences Including Barriers and Facilitators

Participants’ experiences on the feasibility and usability of the VOC were collected during a semistructured interview following the UTAUT2 interview framework. All interviews were audiorecorded and transcribed verbatim using ATLAS.ti 7.1 qualitative data analysis software. In addition, 2 individual researchers (JMJ and T Chau) analyzed transcripts using the thematic content analysis. Interview transcripts were reviewed, coded, and recurrent themes were defined. Eventually, barriers, facilitators, positive effects, and negative effects were identified. Findings were discussed until consensus was achieved. All barriers, facilitators, positive effects, and negative effects were rewritten into general statements and presented according to the UTAUT2 interview framework. We distinguished between factors that affected the VOC use (barriers and facilitators) and effects after use (positive and negative effects).

## Results

In this study, 20 participants installed the required apps and attended an individual 45-minute training session with the eNurse. All participants completed the 6-week study. Table 2 summarizes the basic characteristics of the study population.

### Use and Technical Feasibility

Table 3 presents the agreement between expected and actual user statistics, which is discussed in detail below.

**Table 2:** Participant characteristics (N=20).

Characteristic		n (%)
Age (years), mean (SD), range		43 (3.5), 18-68
**Gender**		
	Female	10 (50)
	Male	10 (50)
**Education level**		
	Senior general secondary education	2 (10)
	Secondary vocational education	7 (35)
	Higher professional education	6 (30)
	University education	5 (25)
**Previous experience self-monitoring related to disease or treatment**		
	Yes	6 (30)
	No	14 (70)

**Table 3:** User statistics per participant compared with the expected user statistics.

Tool or platform	Expected user statistics per participant	Mean or median user statistics per participant	Adherence	
			N	n (%)^a^
Withings blood pressure monitor	10 measurements (2 periods of 5 subsequent days)	13 (IQR^b^: 11-30; range: 5-52) measurements	19	11 (58)
Withings Body+	12 measurements (2 times per week)	23 (SD 2.89, range: 5-42) measurements	19	7 (37)
Q1.6	42 pain scores and 3 completed DLQIs^c^	42 pain scores (IQR: 40-42, range: 35-42); 85% completed all DLQIs	20	14 (70) for the pain scores; 17 (85) for the DLQIs
MedApp	Medication list	2 medication lists complete, 5 incomplete, 5 absent, 8 not linked	—	—
Kanta	Variable	1063 messages in 210 conversations	—	—
FaceTalk	One appointment	One appointment	20	20 (100)
Personal health record	6 log-ins	14 (IQR: 5-19, range: 1-49)	20	13 (65)
Patient portal system	6 log-ins	6 (IQR: 4-14, range: 1-25)	20	12 (60)

^a^This column presents the percentage of participants fully adhering to that part of the protocol (eg, if 11 of 19 participants performed all expected 10 measurements, adherence was 58%).

^b^IQR: interquartile range.

^c^DLQI: Dermatology Life Quality Index.

### Platforms

#### Data Collection

Data collected with 4 of the provided tools were registered in the PHR. In addition, the PPS received real-time data from the 2 Withings devices. The PHR and PPS were consulted by all participants. For the PPS, the median log-ins were 6 (IQR: 4-14; range: 1-25), with a total of 165 log-ins, of which 65.4% (108/165) were by participants from the internal medicine outpatient clinic and 34.5 % (57/165) from the dermatology outpatient clinic. For the PHR, the median log-in frequency was 14 (IQR: 5-19; range: 1-49), with a total number of 378 log-ins, of which 60.8% (230/378) were by participants from the internal medicine outpatient clinic and 39.1% (148/378) from the dermatology outpatient clinic. The actual use of the PHR compared with the expected use ranged from 17% to 817%, and for the PPS, the actual use ranged from 17% to 417%.

All participants were able to link Q1.6 to their PHR. Only 12 of the 20 participants were able to successfully link MedApp to the PHR. Withings devices were successfully connected to the PHR for 16 participants and to the PPS for 17 participants. All, but 1 participant, were able to connect the Withings devices to at least one of the platforms.

For the Withings BPM, measurements occasionally did not show in the PHR or the PPS or only partly (eg, only blood pressure or pulse rate).

**Table 4:** The number of messages per conversation via Kanta, subdivided by theme.

Theme	Total conversations, n (range)	Total messages	Mean messages per conversation, n (range)
Introduction	17 (0-2)	36	2 (1-6)
Study and administration	65 (0-8)	274	4 (1-19)
Functionality tools	60 (0-8)	425	7 (1-36)
Planning and appointments	64 (0-9)	302	5 (1-20)
Medical question	4 (0-1)	26	7 (5-8)

#### Kanta

In total, 1063 messages were sent in 210 conversations by 19 participants. The mean number of messages per conversation was 5. The conversations could be divided by theme (Table 4).

The eNurse initiated 129 conversations and participants 81 conversations. The eNurse started most conversations on “Study and administration” and “Planning and appointments” 48 and 45 times, respectively. The participants initiated most conversations on “Functionality tools” 40 times.

Problems with notifications were experienced, where no notifications would show for new messages. In addition, typing messages had a delay for one participant and concept messages were not saved when closing the app. Kanta was not compatible with all smartphones.

#### Facetalk

All participants had at least one successful FaceTalk appointment with the eNurse, which lasted 10-15 minutes. The adherence was 100%. During the FaceTalk appointments, some technical problems were observed. One participant could not manage to get sound, another participant could not accomplish to switch to the front camera, disconnection was experienced once, and some (older) versions of operating systems were incompatible with FaceTalk. Moreover, one appointment was interrupted by a phone call received by the eNurse.

#### Q1.6

All participants were able to use Q1.6. The median number of days a pain score was entered was 42 (IQR: 40-42; range: 35-42). In this study, 18 of the 20 participants completed >90% of the daily pain scores, and the 2 remaining participants completed 83% and 88% of the daily pain scores. The adherence was 83%-100%.

In addition, 17 of the 20 participants completed all 3 2-weekly DLQIs; 2 participants completed 2 of the 3 DLQIs, and partially completed the remaining DLQI. However, 2 participants did not fill 1 of the 3 DLQIs. The adherence was 67%-100%. Of note, 7 of the participants recruited from the Department of Internal Medicine mentioned that the questions did not relate to their disease, which was perceived as a barrier. Some problems with Q1.6 appeared where the app continued to give notifications after the completion of the questionnaire for 1 participant. Moreover, 1 participant stated that the pop-up appeared at inconvenient times, which resulted in answering without thought.

#### MedApp

In this study, 12 participants were able to connect MedApp with the PHR. Reasons for unsuccessful linking were the absence of connection possibility, incompatible smartphone software, or other unknown reasons. All participants used medication. Overall, 2 of 12 participants filled out MedApp with their complete medication list, and 5 of 12 participants partially filled out MedApp with their medication list. For the remaining participants who linked MedApp with the PHR, it did not show any medication entries, although all participants claimed to have used MedApp during the interview. The adherence was 17% for participants able to make a connection. For 7 participants, the habit of taking medication was already present, making MedApp obsolete. Some other problems were reported where scanning medication did not work properly, and medication compliance was not correctly reported. MedApp was not compatible with all smartphones.

#### Withings Blood Pressure Monitor

The median number of measurements taken was 13 (IQR: 11-30, range: 5-52). One participant was excluded from the analysis because no connection was made between the device and the 2 platforms. Thus, 11 of 19 participants (58%) measured their blood pressure according to the protocol in 2 periods for 5 days, or more often. In addition, 6 of 19 participants measured their blood pressure ≥10 times in 6 weeks, but not in 2 periods of 5 subsequent days. Together, 89% (17/19) measured their blood pressure ≥10 times. According to participants, reasons for the nonadherence were anxiety, technical issues, or when the blood pressure was deemed less relevant for their situation. Participants had different experiences with the Withings BPM: 6 participants reported it was an easy-to-use device, whereas 3 reported taking measurements required precision. Notably, the BPM occasionally reported an error, which indicated a measurement could not be completed after which the participant had to try again. One participant reported anxiety because of the frequency of blood pressure measurements.

#### Withings Body+ Weight Scale

The mean number of measurements taken was 23 (SD 2.89, range: 5-42). One participant was excluded from the analysis because of unsuccessful linking of the device to both platforms. Overall, 37% (7/19) participants measured their weight according to protocol, and 47% (9/19) measured their weight ≥12 times but not in 2 times per week. Thus, 16 of 19 participants measured their weight ≥12 times during the 6-week study.

**Table 5:** Barriers and facilitators mentioned by participants in the user experience interviews for the Virtual Outpatient Clinic.

Variable			Facilitator	Barrier
**Performance expectancy**				
	**Time expectancy**		5	—
		Saves time	3	—
		Accessible at any time	1	—
		Health professionals have real-time access to data	1	—
	**Convenience expectancy**		13	—
		Creates more awareness	4	—
		Quick communication	2	—
		Safe communication	2	—
		Feeling of being in control of own health	2	—
		Less barriers to reach out	1	—
		Sharing information on own initiative	1	—
		Peer-like communication	1	—
**Effort expectancy**				
	**Ease of use**		4	1
		Takes little effort to use	1	—
		Communication requires signing in	—	1
		Clear layout	1	—
		Easy to use	2	—
**Social influence**				
	**Practitioner influence**		1	—
		Inspired by doctor	1	—
	**Peer influence**		1	—
		Inspired by partner	1	—
**Facilitating conditions**				
	**Technology aspects**		—	1
		Not suitable for all smartphones	—	1
	**Security and confidentiality**		1	1
		Everything is digital	—	1
		The hospital is trustworthy	1	—
**Hedonic motivation**				
	**Usage enjoyment**		3	1
		Being aware of health status is fun	1	—
		No priority		1
		Visualization of data is fun	1	—
		The trend line is insightful	1	—
	**Novelty enjoyment**		2	—
		Gadget-factor is fun	2	—

**Table 6:** Positive and negative effects mentioned by participants in the user experience interviews for the Virtual Outpatient Clinic.

Variable			Positive effect	Negative effect
**Performance expectancy**				
	**Time expectancy**		1	—
		Saves time	1	—
	**Convenience expectancy**		11	—
		Creates more awareness	6	—
		Awareness leads to changes in behavior	2	—
		Quick communication	2	—
		A trend line is more insightful than single measurements	1	—
**Facilitating conditions**				
	**Technology aspects**		—	1
		Apps require substantial data storage	—	1
**Hedonic motivation**				
	**Usage enjoyment**		1	—
		Being aware of health status is fun	1	—

### Barriers and Facilitators

In the interviews with individual participants, the user experience of the integral VOC approach was discussed. The interviews lasted between 30 and 90 minutes. Table 5 presents barriers and facilitators for the VOC use, weighed by how often they were mentioned and divided in themes by the UTAUT2 model. Table 6 presents positive and negative effects of the VOC use, weighed by how often they were mentioned and divided in themes by the UTAUT2 model. Multimedia Appendix 3 provides barriers, facilitators, positive effects, and negative effects specific for each tool and platform.

### Participant Suggestions

From the qualitative interviews, participants’ suggestions were obtained to further improve the VOC. Participants expressed the need for a single app or portal that integrates all mHealth tools. The VOC package should be easily installed and linked to a platform because this was perceived as a lot of work by most participants. The instruction on the different aspects of the VOC should be extended. Participants also opted for more tools to add, such as a food diary and a linked glucose-measuring device. Suggestions on the different tools were made as well. For FaceTalk, participants wished for the option to record and store sessions, with the goal to be better able to recall information and decisions that were made. For Kanta, notifications should include a preview of the message and the function to take a screenshot should be added. MedApp should be improved by the ability to prioritize medication. The frequency of reminders should be adjustable. The Q1.6 app needs a “not now” function, and the questionnaires should be based on the disease. Some participants mentioned a preference for the numbered scale to indicate pain instead of a VAS. For Withings, a reminder was thought useful to indicate when measurement should be taken. For the PHR and PPS, some suggestions were also mentioned by participants. It should be indicated what a healthy range is for different health parameters. The platform should also be available as an app. Another request was the addition of imaging results to the platform.

## Discussion

### Principal Findings

In this study, we assessed the feasibility and functionality of a cloud-based VOC. Participants successfully used all features of the VOC and shared a positive attitude toward different self-measurement tools. Although all tools were frequently used, our quantitative and qualitative analyses revealed that technical issues prevented participants from taking measurements on a few occasions. The functionality of the VOC, in general, was well received. Patients stated that better integration of apps and platforms, for instance, in a single smartphone app, would further improve user-friendliness. However, some things need to be discussed first.

The qualitative analysis revealed that the facilitators for using the VOC outweighed the barriers (30 vs 4, respectively). Study participants found that the VOC saved time, was convenient, and easy to use. Traditionally, the burden of going to the hospital is high, as patients have to skip work, travel, pay a parking fee, and spend time waiting. For people with chronic conditions, these visits are often short and can be considered as a regular “check-up.” As a VOC can facilitate the exchange of data, monitoring, and virtual consultations, this new way of delivering care could help in reducing the number of these time-consuming hospital visits.

Although most self-management tools were frequently used and considered by the users, the protocol adherence was lower than expected, primarily for the BPM and weight scale. Besides the technical issues mentioned above, this could be related to the complex measurement schedule and the high number of tools provided in this study. More personalized schedules, allowing users to perform measurements whenever they are ready for it, could further improve adherence. In addition, we found that some tools provided to participants were more interesting for one group than the other. For example, the Q1.6 smartphone app with questions about pain and skin condition was focused on patients of the dermatology outpatient clinic, whereas the BPM was more relevant for patients of the internal medicine outpatient clinic, who often have hypertension. This also emphasizes the need for a personalized set of tools and schedule to make the VOC as useful as possible for both patients and professionals. Although some tools were less relevant to 1 of the 2 groups and the adherence was lower than expected, participants kept using the tools throughout the study period. This is remarkable because we expected a slight decrease in the use of specific tools, as relative advantage is one of the factors negatively influencing the frequency of use [19].

Although this study primarily intended to determine the feasibility and functionality of a multicomponent digital health service, participants reported an increased awareness of their health status as a result of using the service. This could be the first sign of the effectiveness of the provided system. Furthermore, participants asked medical questions via the secured messaging app, whereas the purpose of this app was to discuss the functionality of the tools and report technical problems. This shows that patients are open to using virtual communication tools that are suitable for discussing medical issues.

### Other Research

Empowering patients by giving them an active role in health self-management is not a new concept [40]. The goal of most studies was to determine the effectivity in improving health status; however, the high-quality evidence is lacking to prove the effectiveness [41]. As eHealth and mHealth tools are rarely integrated regarding the data collection and data are often not accessible to patients, scientific studies in this field are lacking. As discussed earlier, most studies on eHealth and mHealth are focused on one specific disease group and do not discuss data sharing and communication between doctors and patients. However, Alnosayan et al provided a multicomponent system for patients [20]. They focused on patients with heart failure and support after discharge. The monitoring system used in their study contained a weight scale, BPM, glucose meter, and a short daily questionnaire. Participants were invited to use the system for 6 months. Their results were comparable to our findings as follows: participants requested a personalized system and integration with other monitoring tools, and visualizations of health data were helpful to gain insight into the health status. Although our study only lasted 6 weeks, and the patient groups between the studies are different, the results show that a VOC is likely to be maintained continuously and will also work for patients with more acute conditions such as presented by Alnosayan et al [20]. The VOC used in this study has the advantage that it is generic and can be personalized for any patient, regardless of their disease or comorbidity.

### Strengths and Limitations

A strength of this study is that we combined different tools and integrated data collection, compared with other studies that focused on a single tool. Another asset is the combination of quantitative and qualitative methods, resulting in a rich dataset with in-depth information and user experiences. However, a limitation of this study is related to the nonrandomized convenience sample, as participants with interest in technology and digitalization are more likely to participate in this study. This may have resulted in an overestimation of positive effects. Another limitation is the relatively short study period, making it impossible to study long-term effects.

### Implications for Practice

Doctors need to be aware of changes in health care regarding eHealth and mHealth. They, but also their patients, could benefit from integrated digital technology such as the VOC. Patients might already track certain health parameters with wearable devices or smartphones that may be valuable to share. Patients need to realize that digital technology facilitates an active role in their health management. Owing to the new possibility to collect data with various devices and tools and store them in a cloud-based platform, including the possibility to connect to the hospital’s EMR, patient-centered care and self-monitoring have become available. Using the VOC in daily practice could potentially result in less frequent physical visits, reduction of overconsumption of care, and a more continuous observation with better prevention and treatment. Experimental study designs to further assess the clinical value of the VOC are needed.

### Conclusions

The VOC is a promising integrated Web-based technology that combines self-management, data sharing, and communication between patients and professionals. The system can be personalized by connecting various numbers of components, which could make it a relevant tool for other patient groups. Before a system, such as the VOC, can be implemented in the daily practice, further integration of all tools into a single app is needed. Moreover, the user-friendliness of different tools should be improved, guided by the wide spectrum of barriers and suggestions mentioned by study participants. Subsequently, a prospective study focused on evaluating outcomes, costs, and patient-centeredness should be conducted.

## Appendix

Multimedia Appendix 1 Screenshot of Patients Know Best (PKB), showing an overview of the measurements performed by a user (example data).

Multimedia Appendix 2 Screenshot of Patients Know Best (PKB), showing a detailed overview of the blood pressure measurements taken by a user (example data).

Multimedia Appendix 3 Specification of barriers, facilitators, positive and negative effects of individual components.

## Abbreviations

BPM: blood pressure monitor
DLQI: Dermatology Life Quality Index
eHealth: electronic health
EMR: electronic medical record
IQR: interquartile range
mHealth: mobile health
PHR: personal health record
PKB: Patients Know Best
PPS: patient portal system
VAS: visual analog scale
VOC: Virtual Outpatient Clinic
UTAUT2: Unified Theory of Acceptance and Use of Technology 2
