# Supplementary figures and images for: A Cloud-Based Virtual Outpatient Clinic for Patient-Centered Care: Proof-of-Concept Study
Source: J Med Internet Res. 2018 Sep 24;20(9):e10135. doi: 10.2196/10135 (PMC6231839; doi:10.2196/10135)

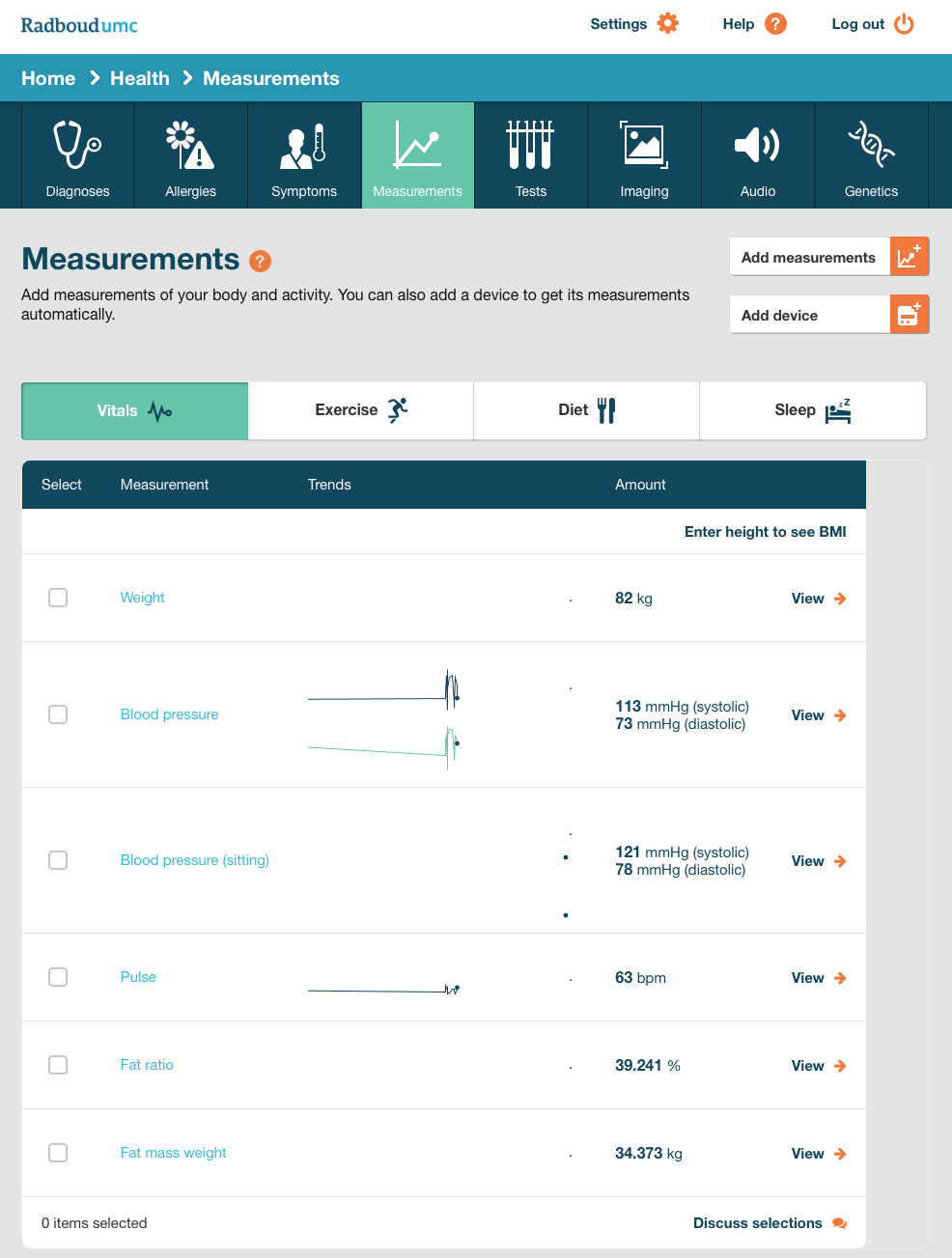

Supplement: Multimedia Appendix 1 [file jmir_v20i9e10135_app1.png]

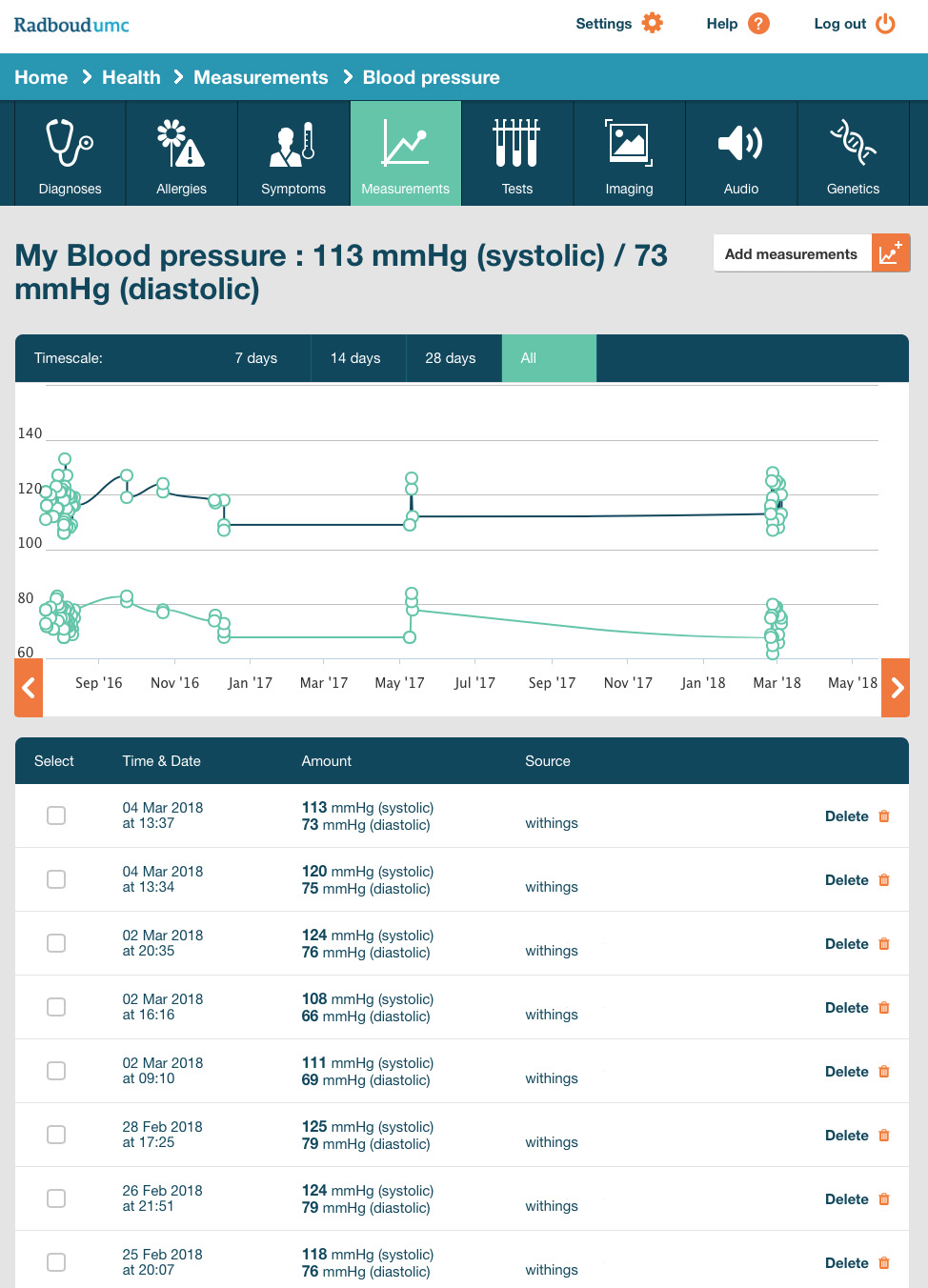

Supplement: Multimedia Appendix 2 [file jmir_v20i9e10135_app2.png]
