# Supplementary material for: A Cloud-Based Virtual Outpatient Clinic for Patient-Centered Care: Proof-of-Concept Study
Source: J Med Internet Res. 2018 Sep 24;20(9):e10135. doi: 10.2196/10135 (PMC6231839; doi:10.2196/10135)
Supplement: Multimedia Appendix 3 [file jmir_v20i9e10135_app3.pdf]

Table 1A. Barriers and facilitators mentioned by patients in the user experience interviews for the use of FaceTalk in the virtual outpatient clinic, weighed by how often they were mentioned. Divided in themes by the UTAUT2 model.

			Facilitator	*Barrier*
Performance expectancy				
	1. Time expectancy		*4*	
		1.1 Saves time and costs	3	
		1.2 Less travelling time	1	
	2. Convenience expectancy		6	*1*
		2.1 It can replace a check-up appointment	3	
		2.2 It is equivalent to a face-to-face conversation	2	
		2.3 Facial expressions are visible	1	
		2.4 No added value to a telephone call		1
Effort expectancy				
	3. Ease of use		*3*	*2*
		3.1 Video calling aids personal feedback	1	
		3.2 Unpleasant/Pleasant way of communication	1	2
		3.3 Easy installation	1	
Facilitating conditions				
	4. Technology aspects		*3*	
		4.1 Technical aspects function well	3	

Table 1B. Positive and negative effects mentioned by patients in the user experience interviews for the use of FaceTalk in the virtual outpatient clinic, weighed by how often they were mentioned. Divided in themes by the UTAUT2 model

			Positive effect	Negative effect
Performance expectancy				
	1. Time expectancy		*4*	
		1.1 Saves time and costs	1	
		1.2 Less travelling time	3	

Table 2. Barriers and facilitators mentioned by patients in the user experience interviews for the use of Kanta in the virtual outpatient clinic, weighed by how often they were mentioned. Divided in themes by the UTAUT2 model.

			Facilitator	Barrier
Performance expectancy				
	1. Time expectancy		*1*	
		1.1 Saves time and costs	1	
	2. Convenience expectancy		*9*	
		2.1 Ability to send a message at any time	4	
		2.2 Ability to confirm or reschedule appointments	3	
		2.3 Ability to send medical photo's	1	
		2.4 The answers are reassuring	1	
Effort expectancy				
	3. Ease of use		*17*	
		3.1 Useful as a helpdesk	5	
		3.2 Easy to use	5	
		3.3 Quick communication	4	
		3.4 No sign-in required	1	
		3.5 It works like traditional messenger apps	1	
	4. Impact on health		*1*	
		4.1 Get a better grasp on personal information	1	
Facilitating conditions				
	5. Technology aspects			*2*
		5.1 Not suitable for all mobile phones		2
	6. Support		*2*	
		6.1 Direct way of communication	2	
	7. Security and confidentiality		*2*	*1*
		7.1 Assured of contact identity	2	
		7.2 It is a holdback to send medical photo's		1

Table 3A. Barriers and facilitators mentioned by patients in the user experience interviews for the use of medApp in the virtual outpatient clinic, weighed by how often they were mentioned. Divided in themes by the UTAUT2 model.

			Facilitator	Barrier
Performance expectancy				
	1. Convenience expectancy		*2*	*8*
		1.1 Habit of taking medication is already present		7
		1.2 Provides a medication overview	1	
		1.3 Provides assurance on already taken medication	1	
		1.4 Not relevant for ointments		1
Effort expectancy				
	2. Ease of use		*3*	*3*
		2.1 Setting and receiving reminders	2	
		2.2 Changing the medication schedule is difficult		1
		2.3 No personal contact		1
		2.4 The pharmacist does not accept orders from MedApp		1
		2.5 Scanning medication is easy	1	
Facilitating conditions				
	3. Technology aspects		*3*	*4*
		3.1 Medication scanner does not work properly		1
		3.2 The app did not work		1
		3.3 Keeps track of the inventory	2	1
		3.4 Medication schedule settings does not work optimally		1
		3.5 Entering medication manually works properly	1	

Table 3B. Positive and negative effects mentioned by patients in the user experience interviews for the use of MedApp in the virtual outpatient clinic, weighed by how often they were mentioned. Divided in themes by the UTAUT2 model.

			Positive effect	Negative effect
Performance expectancy				
	1. Convenience expectancy		*4*	*1*
		1.1 Reminders increase compliance	4	
		1.2 Reminders lose their meaning over time		1
Effort expectancy				
	2. Impact on health		*1*	
		2.1 More consistent use of medication	1	
Facilitating conditions				
	3. Technology aspects			*2*
		3.1 Due to unclear notifications,		2

		medication was taken too late or not at all		

Table 4A. Barriers and facilitators mentioned by patients in the user experience interviews for the use of MijnRadboud in the virtual outpatient clinic, weighed by how often they were mentioned. Divided in themes by the UTAUT2 model.

			Facilitator	*Barrier*
Performance expectancy				
	1. Convenience expectancy		*15*	
		1.1 Overview of the health status	10	
		1.2 Clear and user friendly	3	
		1.3 Ability to reschedule appointments	1	
		1.4 Access to communication between hospital and general practitioner	1	
Effort expectancy				
	2. Ease of use		*8*	
		2.1 Already familiar with the system	5	
		2.2 Appointment reminder by e-mail	1	
		2.3 Ability to ask medical questions	1	
		2.4 Clear lay-out	1	
	3. Impact on health		*1*	
		3.1 The visual overview of data increases awareness	1	
Facilitating conditions				
	4. Technology aspects			*1*
		4.1 Misspellings in the username cannot be corrected		1
	5. Security and confidentiality		*2*	
		5.1 Signing in with two-step verification is safe	2	

Table 4B. Positive and negative effects mentioned by patients in the user experience interviews for the use of MijnRadboud in the virtual outpatient clinic, weighed by how often they were mentioned. Divided in themes by the UTAUT2 model.

			Positive effect	Negative effect
Performance expectancy				
	1. Convenience expectancy		*2*	
		1.1 Overview of the health status	2	
Effort expectancy				
	2. Impact on health use		*1*	
		2.1 The visual overview of data increases awareness	1	

Table 5. Barriers and facilitators mentioned by patients in the user experience interviews for the use of Patients Know Best in the virtual outpatient clinic, weighed by how often they were mentioned. Divided in themes by the UTAUT2 model.

			Facilitator	*Barrier*
Performance expectancy				
	1. Convenience expectancy		*4*	
		1.1 Sharing information on own initiative	2	
		1.2 Medical information is shown in clear graphs	1	
		1.3 More control over personal information	1	
Effort expectancy				
	2. Ease of use		*4*	*1*
		2.1 Lay-out was intuitive	2	
		2.2 Lay-out was not intuitive		1
		2.3 Signing in without two-step verification	2	
Facilitating conditions				
	3. Technology aspects		*1*	
		3.1 Ability to add own data	1	
	4. Security and confidentiality			*1*
		4.1 No option to delete all data		1

Table 6A. Barriers and facilitators mentioned by patients in the user experience interviews for the use of Q1.6 in the virtual outpatient clinic, weighed by how often they were mentioned. Divided in themes by the UTAUT2 model.

			Facilitator	*Barrier*
Performance expectancy				
	1. Convenience expectancy		*1*	*10*
		1.1 Helps with self-monitoring	1	
		1.2 The extended questionnaire is unrelated to the disease		7
		1.3 The extended questionnaire is not useful		2
		1.4 No option to return to a previous question		1
Effort expectancy				
	2. Ease of use		*3*	*2*
		2.1 Takes little effort to use	3	
		2.2 Loading the app takes long		1
		2.3 Unable to postpone questions at inconvenient moments		1
Facilitating conditions				
	3. Technology aspects		*2*	*2*
		3.1 Not suitable for all mobile phones		1
		3.2 Pain score rating system	2	
		3.3 The extended questionnaire does not work properly		1
	4. Prompts		*4*	*2*
		4.1 Repeated reminders	4	2
Hedonic motivation				
	5. Usage enjoyment		*1*	
		5.1 Reminds to self-care	1	

Table 6B. Positive and negative effects mentioned by patients in the user experience interviews for the use of Q1.6 in the virtual outpatient clinic, weighed by how often they were mentioned. Divided in themes by the UTAUT2 model.

			Positive effect	Negative effect
Performance expectancy				
	1. Convenience expectancy		*1*	
		1.2 Professional feedback on data	1	
Hedonic motivation				
	2. Usage enjoyment		*4*	
		2.1 Reminds to self-care	4	

Table 7A. Barriers and facilitators mentioned by patients in the user experience interviews for the use of Withings in the virtual outpatient clinic, weighed by how often they were mentioned. Divided in themes by the UTAUT2 model.

			Facilitator	*Barrier*
Performance expectancy				
	1. Convenience expectancy		*4*	*1*
		1.1 Creates awareness on health status	4	
		1.2 Blood pressure measurement is unrelated to the disease(B)		1
Effort expectancy				
	2. Ease of use		*25*	*4*
		2.1 Devises work intuitively	10	
		2.2 Measurements are easily and quickly performed(B)	6	
		2.3 Measurements are easily and quickly performed(A)	4	
		2.4 Blood pressure measurements needs to be performed precisely(B)		3
		2.5 Small burden	2	
		2.6 Blood pressure monitor often fails to work(B)		1
		2.7 The trend line is insightful	1	
		2.8 The device shows why a measurement fails	1	
		2.9 The app is easy to use	1	
Facilitating conditions				
	3. Technology aspects		*1*	*3*
		3.1 Unclear cause of failing measurements		1
		3.2 Scale occasionally does not work properly(A)		1
		3.3 Results are deviant		1
		3.4 Blood pressure monitor averages three measurements	1	
	4. Security and confidentiality		*1*	
		4.1 The hospital is trustworthy	1	
Hedonic motivation				
	5. Usage enjoyment		*11*	
		5.1 Fun to keep track	2	
		5.2 Fun to do a measurement	1	
		5.3 Motivating e-mails	2	
		5.4 Scale shows the weather forecast(A)	2	
		5.5 Attractive lay-out	2	
		5.6 Awareness leads to changes in behavior	2	

Statements refer to (A) weight scale, (B) blood pressure monitor.

Table 7B. Positive and negative effects mentioned by patients in the user experience interviews for the use of Withings in the virtual outpatient clinic, weighed by how often they were mentioned. Divided in themes by the UTAUT2 model.

			Positive effect	Negative effect
Performance expectancy				
	1. Time expectancy		*3*	
		1.1 Self-monitoring saves time	3	
	2. Convenience expectancy		*10*	
		2.1 Creates awareness on health status	5	
		2.2 Awareness leads to changes in behavior	3	
		2.3 Home measurements show a more reliable overview	2	
Effort expectancy				
	3. Ease of use		*1*	
		3.1 Measurements are easily and quickly performed$^{(B)}$	1	
Hedonic motivation				
	4. Usage enjoyment		*1*	*1*
		4.1 Daily measuring causes anxiety		1
		4.2 Motivating e-mails	1	

Statements refer to (A) weight scale, (B) blood pressure monitor.

Table 8A. Barriers and facilitators mentioned by patients in the user experience interviews for the virtual outpatient clinic, weighed by how often they were mentioned. Divided in themes by the UTAUT2 model.

			Facilitator	*Barrier*
Performance expectancy				
	1. Time expectancy		*5*	
		1.1 Saves time	3	
		1.2 Accessible at any time	1	
		1.3 Health professionals have real-time access to data	1	
	2. Convenience expectancy		*13*	
		2.1 Creates more awareness	4	
		2.2 Quick communication	2	
		2.3 Safe communication	2	
		2.4 Feeling of being in control of own health	2	
		2.5 Less barriers to reach out	1	
		2.6 Sharing information on own initiative	1	
		2.7 Peer-like communication	1	
Effort expectancy				
	3. Ease of use		*4*	*1*
		3.1 Takes little effort to use	1	
		3.2 Communication requires signing in		1
		3.3 Clear lay-out	1	
		3.4 Easy to use	2	
Social influence				
	4. Practitioner influence		*1*	
		4.1 Inspired by doctor	1	
	5. Peer influence		*1*	
		5.1 Inspired by partner	1	
Facilitating conditions				
	6. Technology aspects			*1*
		6.1 Not suitable for all mobile phones		1
	7. Security and confidentiality		*1*	*1*
		7.1 Everything is digital		1
		7.2 The hospital is trustworthy	1	
Hedonic motivation				
	8. Usage enjoyment		*3*	*1*
		8.1 Being aware of health status is fun	1	
		8.2 No priority		1
		8.3 Visualization of data is fun	1	
		8.4 The trend line is insightful	1	
	9. Novelty enjoyment		*2*	
		9.1 Gadget-factor is fun	2	

Table 8B. Positive and negative effects mentioned by patients in the user experience interviews for the virtual outpatient clinic, weighed by how often they were mentioned. Divided in themes by the UTAUT2 model.

			Positive effect	Negative effect
Performance expectancy				
	1. Time expectancy		*1*	
		1.1 Saves time	1	
	2. Convenience expectancy		*11*	
		2.1 Creates more awareness	6	
		2.2 Awareness leads to changes in behavior	2	
		2.3 Quick communication	2	
		2.4 A trend line is more insightful than single measurements	1	
Facilitating conditions				
	3. Technology aspects			*1*
		3.1 Apps require substantial data storage		1
Hedonic motivation				
	4. Usage enjoyment		*1*	
		4.1 Being aware of health status is fun	1	
